# Anti-GAPDH Autoantibody Is Associated with Increased Disease Activity and Intracranial Pressure in Systemic Lupus Erythematosus

**DOI:** 10.1155/2019/7430780

**Published:** 2019-03-31

**Authors:** Jingjing Sun, Xue Li, Haotian Zhou, Xiaoyun Liu, Jingjing Jia, Qizhi Xie, Sijia Peng, Xiaolin Sun, Qingwen Wang, Li Yi

**Affiliations:** ^1^Department of Neurology, Peking University Shenzhen Hospital, Shenzhen, China; ^2^Department of Rheumatology and Immunology, Peking University People's Hospital, Beijing, China; ^3^Institute of Analytical Chemistry and Synthetic and Functional Biomolecules Center, College of Chemistry and Molecular Engineering, Peking University, Beijing, China; ^4^Department of Rheumatology and Immunology, Peking University Shenzhen Hospital, Shenzhen, China

## Abstract

**Objective:**

Systemic lupus erythematosus (SLE) is an immune disease characterized by multiorgan involvement. Neuropsychiatric systemic lupus erythematosus (NPSLE) is one of the most devastating complications of SLE, which lacks efficient diagnostic biomarkers. The recent studies on the anti-GAPDH autoantibodies suggested its potential pathogenic roles in NPSLE. However, the clinical relevance of the anti-GAPDH autoantibodies in patients with SLE is still elusive. In this study, we sought to determine the serum levels of the anti-GAPDH autoantibodies in patients with SLE to investigate the clinical significance of the anti-GAPDH autoantibodies in SLE.

**Methods:**

Concentrations of the glyceraldehyde 3-phosphate dehydrogenase autoantibodies (anti-GAPDH autoantibodies) in the serum of 130 SLE patients and 55 healthy individuals were determined by enzyme-linked immunosorbent assay (ELISA). Among the 130 SLE patients, 95 were SLE patients without neuropsychiatric symptoms and 35 had NPSLE. White blood cell (WBC) count, hemoglobin (HB), platelet count (PLT), IgG, IgA, IgM, anti-dsDNA, C3, C4, erythrocyte sedimentation rate (ESR), C-reactive protein (CRP), RF, anti-cardiolipin (Acl), ANA, AnuA, anti-SSA, anti-SSB, *β*2-GPI, urinalysis, and 24 h urine protein were measured by standard laboratory techniques. Systemic lupus erythematosus disease activity index 2000 (SLEDAI-2K) and Systemic Lupus International Collaborating Clinics/American College of Rheumatology (SLICC/ACR) damage index scores were evaluated accordingly.

**Results:**

The serum levels of the anti-GAPDH autoantibodies were significantly elevated in the SLE patients, especially in the patients with NPSLE (*P* = 0.0011). Elevated serum anti-GAPDH was correlated with increased SLEDAI-2K (*P* = 0.017), ESR, IgG, and IgM and associated with increased intracranial pressure and incidence of cerebrovascular lesions, but it was protective for seizure disorder incidence.

**Conclusions:**

Serum anti-GAPDH autoantibody was increased in both groups of SLE patients with or without neuropsychiatric symptoms and associated with disease severity. It could become an indicator of tissue damages in the brain for the future clinical practice.

## 1. Introduction

Systemic lupus erythematosus (SLE) is a prototypic autoimmune disease characterized by a broad spectrum of autoantibodies and multiorgan involvement, mainly affecting skin, joint, kidney, lung, and nervous system [1, 2]. Neuropsychiatric SLE (NPSLE) is one of the most fatal manifestations of SLE [2]. The recent studies reported that the prevalence of NPSLE manifestations was up to 56% in patients with SLE and the neuropsychiatric damage negatively affected the overall 5-year survival [3, 4]. The manifestations of NPSLE vary from patient to patient and lead to poor prognosis because of clinical heterogeneity and inefficient diagnosis [5]. To date, because of lack of specific diagnostic biomarkers, NPSLE is still diagnosed based on a comprehensive conception of clinical observations, laboratory tests, and imaging techniques [6].

The 1999 ACR-NPSLE case definition including nineteen central and peripheral nervous system clinical manifestations has been widely applied in clinical practice; however, few specific biomarkers have been proved to be efficient enough to diagnose NPSLE timely [7]. Significant correlation has been identified between a series of autoantibodies and NPSLE incidence, such as antiphospholipid antibodies, anti-ribosomal P antibodies, and anti-DNA/NR2 antibodies [5, 8, 9]. However, none of these NPSLE-related autoantibodies has been widely accepted as a specific biomarker for NPSLE diagnosis [7].

Takasaki et al. first reported that GAPDH was one of the elements of proliferating cell nuclear antigens specifically reactive with serum from SLE patients [10]. Of note, Delunardo et al. further identified that the anti-GAPDH autoantibodies specifically reacted with neuronal cells and were associated with cognitive dysfunction in patients with SLE [11]. However, the clinical relevance and pathogenic roles of the anti-GAPDH autoantibodies were still elusive. In this study, we determined the serum levels of the anti-GAPDH autoantibodies in a large group of SLE patients, to investigate the clinical association of the anti-GAPDH autoantibodies with NPSLE- and SLE-related clinical manifestations and laboratory features. We found that the anti-GAPDH autoantibodies were significantly increased in patients with SLE, especially in those who developed NPSLE manifestations. The anti-GAPDH autoantibodies were associated with increased SLE disease activity and inflammation as well as increased intracranial pressure, which suggested that it could become a novel biomarker to evaluate the NPSLE activity.

## 2. Materials and Methods

### 2.1. Patients and Samples

In this study, 130 SLE patients were recruited from Department of Rheumatology and Immunology, Peking University People's Hospital, Beijing, China, from June 2017 to May 2018. All patients conformed to the 1997 revised classification criteria of the American College of Rheumatology [12]. Among them, 95 patients were diagnosed as having SLE without neuropsychiatric manifestations (non-NPSLE group) and 35 patients were diagnosed as having NPSLE (NPSLE group) according to the 1999 ACR criteria [7]. 55 age- and sex-matched healthy controls (HC) were recruited from the health examination center of the same hospital. The characteristics of patients and healthy controls at the time of recruitment were listed in Table 1. The study was approved by the Ethics Committee of Peking University People's Hospital according to the Declaration of Helsinki with the reference number 81601417. All participants were informed and signed the consent for this study. The serum sample from each participant was kept frozen at -80°C prior to use.

### 2.2. Clinical and Laboratory Evaluations

The SLE patients' clinical manifestations and laboratory examinations were recorded, which included age, gender, disease duration, white blood cell (WBC) count, hemoglobin (Hb), platelet count (PLT), D-dimer, immunoglobulin G (IgG), immunoglobulin A (IgA), immunoglobulin M (IgM), complement component 3 (C3), complement component 4 (C4), erythrocyte sedimentation rate (ESR), rheumatoid factor (RF), C-reactive protein (CRP), anti-double-stranded DNA antibody (anti-dsDNA Ab), anti-nucleosome antibodies (AnuA), anti-cardiolipin antibody (Acl Ab), anti-SSA antibody (SSA), anti-SSB antibody (SSB), anti-nuclear antibodies (ANA), anti-*β*2 glycoprotein I antibodies (*β*2-GPI Abs), urinalysis, and 24 h urine protein. Systemic lupus erythematosus disease activity index 2000 (SLEDAI-2K) scores were evaluated according to the previous report [13]. Fever was defined as a temperature (*T*) over 38.0°C. Headache was defined according to the ACR definition of lupus headache [14]. Leukocytes and platelets less than 3.5 × 10^9^/L and 100 × 10^9^/L were defined as leukopenia and thrombocytopenia, respectively. Complement C3 less than 0.88 g/L and C4 less than 0.16 g/L were regarded as decreased C3 and C4, respectively. Moreover, anti-dsDNA Abs more than 25 IU/mL or AnuA more than 20 RU/mL were considered as positive. 24 h urine protein more than or equal to 0.5 g/day was defined as proteinuria. The NPSLE patients' clinical manifestations and laboratory examinations were also recorded, which included central and peripheral nervous system manifestations, cerebrospinal fluid (CSF) examination results, and radiologic data of the CNS by brain magnetic resonance imaging (MRI) and/or computed tomography (CT) scan. We also scored the NPSLE patients with the Systemic Lupus International Collaborating Clinics/American College of Rheumatology (SLICC/ACR) damage index according to the previous report [15].

### 2.3. Measurement of the Anti-GAPDH Autoantibodies

Serum levels of the anti-GAPDH autoantibodies were determined by indirect ELISA as previously described [16]. Briefly, 96-well polysorp plates (Nunc, Denmark) were coated with recombinant human GAPDH protein (OriGene, Beijing, China) of 1 *μ*g/mL in carbonate buffer at 4°C overnight. The wells were then washed 4 times with phosphate-buffered saline containing 0.05% Tween-20 (PBS-T) at room temperature and blocked with 3% albumin bovine V (BSA) for 2 hours at 37°C. Serum samples were diluted with PBS-T containing 1% BSA at 1 : 100 and were then added to 96-well plates. After incubation for 1 hour at 37°C, the wells were washed by PBS-T for five times. Then, 100 *μ*L of goat anti-human IgG conjugated to peroxidase, diluted at 1 : 3000, was added to each well and incubated for 1 hour at 37°C. After washing with PBS-T four times, 100 *μ*L of tetramethylbenzidine (NeoBioscience) was added to each well and incubated at 37°C and the reaction was stopped by adding 50 *μ*L of 2.5 M sulfuric acid to each well. Plates were read by a plate reader (BioTek) at an absorbance wavelength of 450 nm optical density (OD 450). Each serum sample was tested in duplicate. For nonspecific background determination, wells coated with recombinant human GAPDH protein were filled only by PBS-T containing 1% BSA instead of serum samples diluted with PBS-T containing 1% BSA, and the rest of the ELISA steps were all the same as above. The values of OD of the anti-GAPDH autoantibodies were transformed to arbitrary units (AU), calculated as follows:

(1) $$\begin{array}{c} \text{AU}=\frac{{\left[{\text{OD}}_{\text{protein}}-{\text{OD}}_{\text{nonspecific background}}\right]}_{\text{test serum}}}{{\left[{\text{OD}}_{\text{protein}}-{\text{OD}}_{\text{nonspecific background}}\right]}_{\text{positive control serum}}}\times 100. \end{array}$$

### 2.4. Statistical Analysis

SPSS13.0 for Windows and GraphPad Prism 5 were used to analyze the data. The distribution of numerical data was expressed by the Shapiro-Wilk test. Numerical data with normal distribution and nonnormal distribution were presented as mean ± standard deviation and median (range), respectively. Statistical significance between the two groups was assessed with the nonparametric Mann-Whitney test, *t*-test, and *χ* ^2^ test. Spearman's rank correlation coefficient was applied to calculate the correlations. The cut-off value of levels of the anti-GAPDH autoantibodies was determined by receiver operating characteristic (ROC) curve analysis. A *P* value less than 0.05 was considered to be statistically significant.

## 3. Results

### 3.1. Characteristics of SLE Patients and Controls

A total of 130 SLE patients and 55 healthy controls were recruited in this study. The total SLE patients were divided into 2 groups based on the presence of neuropsychiatric symptoms: the non-NPSLE group consisted of 95 SLE patients without neuropsychiatric syndromes and the NPSLE group included all the NPSLE patients. The clinical and laboratory characteristics of the patients and controls were summarized in Table 1. The mean age of 130 SLE patients was 36.55 ± 13.90 ranging from 15 to 76 years, and the mean disease duration was 4 years ranging from 12 days to 40 years.

### 3.2. Elevated Serum Levels of the Anti-GAPDH Autoantibodies in SLE Patients

**Figure 1:**
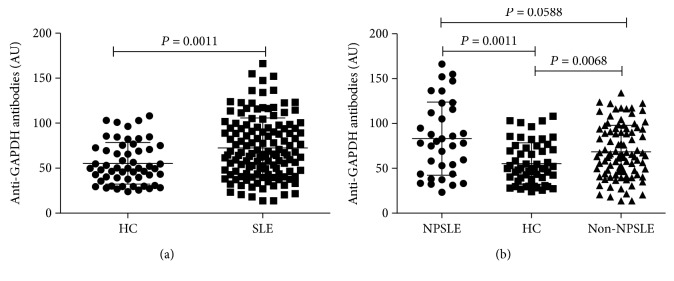
Comparison of the serum levels of anti-GAPDH autoantibodies between SLE patients and HC. (a) Serum anti-GAPDH autoantibodies were higher in total SLE patients versus HC. (b) Serum anti-GAPDH autoantibodies were significantly increased in NPSLE patients and in non-NPSLE patients versus HC.

We firstly compared the serum levels of the anti-GAPDH autoantibodies in healthy controls to those in patients with SLE. As shown in Figure 1(a), the serum levels of the anti-GAPDH autoantibodies in SLE patients (AU: 66.71 (45.83 to 94.50)) were significantly increased compared to healthy controls (AU: 49.91 (37.73 to 72.56)) (*P* = 0.0011). When SLE patients were divided into the NPSLE group and non-NPSLE group, we observed that the anti-GAPDH autoantibody levels in the NPSLE group (AU: 83.07 (43.66 to 115.8)) were more elevated than those in the non-NPSLE group (AU: 68.46 (46.48 to 93.81)) with marginal difference (*P* = 0.0588) (Figure 1(b)). When we compared the serum anti-GAPDH autoantibody levels between the non-NPSLE patients and healthy control, there was also a significant increase of the anti-GAPDH autoantibodies in these SLE patients without neuropsychiatric symptoms (*P* = 0.0068) (Figure 1(b)). These results indicated that the anti-GAPDH autoantibodies were indeed associated with SLE development, in particular associated with NPSLE development.

### 3.3. Elevated Anti-GAPDH Was Associated with SLE Disease Activity

**Figure 2:**
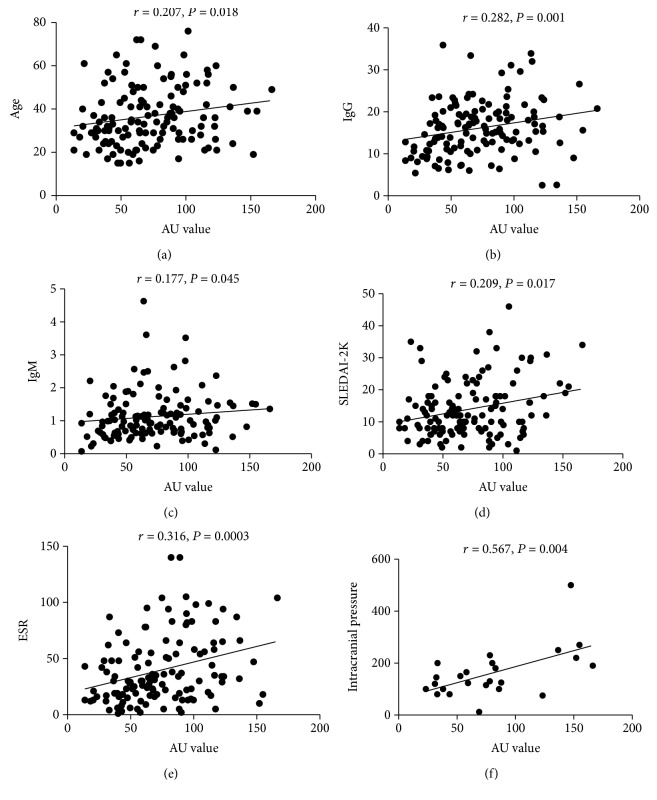
Correlation of the serum level of anti-GAPDH autoantibodies with laboratory parameters in SLE and in NPSLE patients: (a) correlation between the anti-GAPDH autoantibodies and age, (b) correlation between the anti-GAPDH autoantibodies and IgG, (c) correlation between the anti-GAPDH autoantibodies and IgM, (d) correlation between the anti-GAPDH autoantibodies and systemic lupus erythematosus disease activity index 2000 (SLEDAI-2K), (e) correlation between the anti-GAPDH autoantibodies and ESR, and (f) correlation between the anti-GAPDH autoantibodies and intracranial pressure in NPSLE patients.

As shown in Table 2, it was found that the elevated anti-GAPDH autoantibodies were correlated with increased SLEDAI-2K (*r* = 0.209, *P* = 0.017, Figure 2(d) and Table 2). Meanwhile, the elevated anti-GAPDH autoantibodies were also correlated with increased ESR in SLE patients (Figure 2(e) and Table 2). It was also found that the anti-GAPDH autoantibodies were associated with increased serum IgG (*r* = 0.282, *P* = 0.001, Figure 2(b) and Table 2) and IgM (*r* = 0.177, *P* = 0.045, Figure 2(c) and Table 2) levels and decreased incidence of lupus nephritis (Figures 2(b) and 2(c) and Table 3). Anti-GAPDH autoantibodies were also weakly correlated with increasing ages of the SLE patients (Figure 2(a) and Table 2). When SLE patients were grouped into the anti-GAPDH elevated group (AU ≥ 55.09, *n* = 86) and anti-GAPDH normal group (AU < 55.09, *n* = 44) by the cut-off value (AU = 55.09) produced by ROC analysis, it was also found that the anti-GAPDH autoantibodies were associated with the increased inflammation markers ESR and CRP (Table 3). These results suggested that SLE patients with the elevated anti-GAPDH autoantibodies were in more active disease status and the anti-GAPDH autoantibodies might be involved in active inflammation in SLE.

### 3.4. Anti-GAPDH Was Associated with Neuropsychiatric Symptoms in NPSLE Patients

As the anti-GAPDH autoantibody had been proved to be a potential biomarker and pathogenic molecular for NPSLE, we evaluated the clinical relevance of anti-GAPDH with neuropsychiatric manifestations and laboratory features in NPSLE patients. The serum levels of the anti-GAPDH autoantibodies were significantly correlated with elevated intracranial pressure (*r* = 0.567, *P* = 0.004) (Figure 2(f), Supplementary Table 1). Anti-GAPDH autoantibodies were also marginally correlated with increased ESR (*r* = 0.323, *P* = 0.066) and serum IgM (*r* = 0.306, *P* = 0.078) in NPSLE patients (Table 3), similar to the result from the whole SLE patients. When NPSLE patients were grouped into the anti-GAPDH elevated group (AU ≥ 55.09, *n* = 24) and anti-GAPDH normal group (AU < 55.09, *n* = 11) by the cut-off value (AU = 55.09) produced by ROC analysis in Supplementary Table 2, it was also found that the anti-GAPDH autoantibodies were associated with increased incidence of cerebrovascular lesions (*P* = 0.034) but decreased occurrence of seizure disorders (*P* = 0.041, Supplementary Table 2). To further evaluate the connection of anti-GAPDH and brain damage, we applied the SLICC-ACR damage index as a comparator (Supplementary Figure 1). There was a trend of the elevation of serum anti-GAPDH in NPSLE patients with higher SLICC-ACR scores (in the group whose SLICC-ACR score is 3), but no statistical significance is observed between these groups (Supplementary Figure 1(a)). We further compared the SLICC-ACR scores between the anti-GAPDH elevated group and anti-GAPDH normal group (Supplementary Figure 1(b)). Although NPSLE patients with elevated serum anti-GAPDH showed a higher SLICC-ACR scores than patients with normal anti-GAPDH, no statistical significance is achieved (Supplementary Figure 1(b)), which might be due to the limited sample size of NPSLE cohort in this study. These results suggested that NPSLE patients with the elevated anti-GAPDH autoantibodies were in more active disease status and the anti-GAPDH autoantibodies might be involved in specific NPSLE symptom development.

The relationship between the anti-GAPDH autoantibodies and NPSLE-related antiphospholipid antibodies was analyzed. The antiphospholipid antibodies included the lupus anticoagulant, the anti-cardiolipin antibodies, and anti-*β*2 glycoprotein I antibodies. The results showed that the serum levels of the anti-GAPDH autoantibodies were not correlated with the antiphospholipid antibodies or any of its components (*P* > 0.05) (Supplementary Table 1). We also evaluated the incidence of NPSLE in SLE patients positive or negative for the antiphospholipid antibodies. The SLE patients positive for the antiphospholipid antibodies or the anti-cardiolipin antibodies (Acl) showed almost twofold higher prevalence of NPSLE and epilepsy than patients negative for the antiphospholipid antibodies but without statistical significance (Supplementary Tables 3 and 4). The association with cerebrovascular symptom of the anti-GAPDH autoantibodies and the antiphospholipid antibodies was also compared. Cerebral vascular lesions (CVL) were evaluated with brain magnetic resonance imaging (MRI), and most of the CVL were cerebral infarction. Serum anti-GAPDH autoantibodies were significantly associated with CVL (*P* < 0.05), while no such association was found with the antiphospholipid antibodies (*P* > 0.05) (Supplementary Tables 2–4). Increase of epilepsy was found in patients positive for the antiphospholipid antibodies; however, patients with increased anti-GAPDH showed significant decreased incidence of epilepsy (Supplementary Tables 2–4). The absence of correlation between the anti-GAPDH and antiphospholipid antibodies and different association with NPSLE symptoms of these two NPSLE-related autoantibodies implicated that they might play different pathogenic roles in NPSLE development.

## 4. Discussion

According to the previous reports, GAPDH was identified as a target antigen reacting with the serum from patients with autoimmune diseases, such as SLE, Behçet's disease (BD), dermatomyositis (DM), rheumatoid arthritis (RA), and Takayasu's arteritis (TA) [17, 18]. Anti-GAPDH autoantibodies were prevalent in 47% of SLE patients [11]. However, the clinical significance of the anti-GAPDH autoantibodies in SLE was still unclear. Takasaki et al. reported that GAPDH was one of the possible proteins that might have a role in the induction of the autoimmune response and first identified the nuclear localization of GAPDH detected by autoantibodies in SLE serum [10]. More importantly, they suggested that the anti-GAPDH autoantibodies were also important serological markers for SLE [11, 19, 20]. A recent study identified that the anti-GAPDH autoantibodies specifically reacted with neuronal cells and had a significant positive correlation with cognitive dysfunction in patients with SLE [10].

In this study, we determined the serum levels of the anti-GAPDH autoantibodies in SLE patients and healthy controls, and for the first time, we analyzed the clinical relevance of the serum anti-GAPDH levels with laboratory and clinical features of SLE patients. Our results showed that the serum anti-GAPDH levels of SLE patients were significantly higher than those of healthy people. Anti-GAPDH autoantibodies were positively correlated with SLEDAI-2K, ESR, IgG, and IgM, which suggested that anti-GAPDH could work as an indicator of lupus disease activity.

The previous study has revealed a link between the autoantibodies and neuropsychiatric disorders in SLE [21]. Takasaki et al. and Delunardo et al. had proposed GAPDH as a novel autoantigen, and it is expressed in neuronal cells and recognized by the serum autoantibodies from patients with SLE [10, 11]. Our study showed that anti-GAPDH was significantly correlated with increased intracranial pressure in NPSLE patients. Increased intracranial pressure as a premonitory manifestation of NPSLE is indicative of potential brain tissue involvement. Headache and 66.67% of the main NPSLE symptoms are characterized by increased intracranial pressure. The previous study reported that abnormally elevated intracranial pressure was associated with SLE [22], and the causes of increased intracranial pressure included venulitis, aseptic meningitis, immune complex deposition, and/or microocclusion of arachnoid villi [23]. Increased intracranial pressure may be the result of increased venous pressure, increased arachnoid resistance, increased CSF production, or blockage of free CSF flow [23]. Therefore, anti-GAPDH might become a novel indicator of brain tissue damage due to its close correlation with increased intracranial pressure. The previous studies have revealed that increased intracranial pressure was associated with cerebrovascular lesions (CVL) [24, 25]. We also found that the anti-GAPDH autoantibodies were positively associated with cerebrovascular lesions for the first time. When blood-brain barrier (BBB) is compromised in SLE development, autoantibody-mediated neuronal or vascular injury is important in initiating NPSLE symptoms [26]. It is possible that the elevated anti-GAPDH autoantibodies target the GAPDH expressing in the neuronal cells. This possibility could lead to neuron destruction and pathological changes in the brain tissues of NPSLE patients, further resulting in cerebrovascular complications and elevated intracranial pressure. It is of clinical importance to identify the risk factors of NPSLE to provide prognostic insights. These results suggested that NPSLE patients with the elevated anti-GAPDH autoantibodies were in more active disease status and the anti-GAPDH autoantibodies might be associated with cerebrovascular risk in NPSLE.

The clinical manifestations are highly heterogeneous in NPSLE, and the precise pathophysiology of NPSLE is not clearly understood. One possible explanation is that the variety of autoantibodies involved in NPSLE development might play different pathogenic roles due to their diverse cellular or tissue target components, which may activate downstream signaling cascades, resulting in the expression of cytokines and chemokines which contributes to the development of NPSLE symptoms [27] and leads to various associations between the autoantibodies and NPSLE manifestations. For example, the antiphospholipid (aPL) autoantibodies are related to stroke and transverse myelitis, the anti-ribosomal P antibodies are specifically associated with psychosis, and anti-GAPDH is reported to be associated with cognitive dysfunction [11, 28, 29]. In this study, we revealed that elevated anti-GAPDH was positively associated with increased intracranial pressure and cerebrovascular lesion onset; however, significantly lower incidence of seizure disorders was observed in patients with a higher anti-GAPDH, which implicated the complex and differential pathological roles of autoantibodies in SLE development. To date, 20 autoantibodies (eleven brain-specific Abs and nine systemic Abs) associated with NPSLE have been identified [30, 31]. Experimental evidences revealed that autoantibodies reactive with brain antigens acted as key factors in the NPSLE pathogenesis [31]. The underlying pathology by which autoantibodies contribute to the development of NPSLE is still elusive and in need of further studies.

There are some limitations in this study. Although the anti-GAPDH autoantibodies are significantly elevated in the NPSLE group and SLE group, the overlap between groups is so great that the diagnostic significance of this antibody is limited in our clinical practice. It is necessary for us to improve the detection technique of this autoantibody in the future work, which may help improve the feasibility to apply this antibody as a diagnostic or prognostic biomarker in NPSLE diagnosis.

In conclusion, our study demonstrated that the serum anti-GAPDH autoantibodies in patients with NPSLE were more frequently detected than those in SLE patients without NPSLE manifestations and healthy controls. The clinical significance of anti-GAPDH in SLE patients and NPSLE subpopulation was systemically analyzed for the first time, and anti-GAPDH was identified to be the first SLE-related autoantibody significantly associated with increased intracranial pressure, which implicated its potential roles in brain tissue damage induction. Further investigation is needed to determine the exact pathogenic mechanism of anti-GAPDH in NPSLE.

## Figures and Tables

**Table 1:** Characteristics of 130 SLE patients and 55 healthy controls.

	SLE	Control	*t*/*u*/*χ* ^2^	*P*
Age	36.55 ± 13.90	36.13 ± 9.491	0.230	0.818
Gender (F : M)	120 : 10	49 : 6	0.506	0.477
WBC	5.300 (3.638, 7.710)	—	—	—
HB	109.1 ± 20.46	—	—	—
PLT	162.0 (115.0, 237.5)	—	—	—
D-dimer	248.0 (106.0, 658.0)	—	—	—
IgG	15.90 (11.55, 19.85)	—	—	—
IgA	2.480 (1.770, 3.860)	—	—	—
IgM	0.9710 (0.634, 1.415)	—	—	—
C3	0.5970 (0.427, 0.840)	—	—	—
C4	0.1230 (0.062, 0.184)	—	—	—
ESR	30.00 (17.00, 56.00)	—	—	—
RF	30 (23.62%)	—	—	—
CRP	4.750 (1.903, 12.28)	—	—	—
CK	28.50 (18.25, 45.75)	—	—	—
Cr	53.00 (45.00, 66.00)	—	—	—
Anti-dsDNA	46.65 (10.20, 182.3)	—	—	—
AnuA	33.76 (6.948, 164.5)	—	—	—
Anti-SSA	53.91%	—	—	—
Anti-SSB	11.72%	—	—	—
Anti-RNP	49.61%	—	—	—
ANA ≥ 1 : 320	71.09%	—	—	—
SLEDAI-2K ≥ 10	64.62%	—	—	—

F: female; M: male; WBC: white blood cell; HB: hemoglobin; PLT: platelet count; IgG: immunoglobulin G; IgA: immunoglobulin A; IgM: immunoglobulin M; C3: complement component 3; C4: complement component 4; ESR: erythrocyte sedimentation rate; RF: rheumatoid factor; CRP: C-reaction protein; CK: creatine kinase; Cr: creatinine; anti-dsDNA: anti-double-stranded DNA antibodies; AnuA: anti-nucleosome antibodies; anti-SSA: anti-SSA antibody; anti-SSB: anti-SSB antibody; anti-RNP Abs: anti ribonucleoprotein (RNP) antibodies; ANA: anti-nuclear antibodies; SLEDAI-2K: systemic lupus erythematosus disease activity index 2000.

**Table 2:** Correlation of serum anti-GAPDH antibodies with clinical and laboratory features of SLE patients.

Clinical manifestations and laboratory features	Anti-GAPDH	
	Spearman *r*	*P*
Age	0.207	**0.018**
WBC	-0.048	0.588
HB	-0.134	0.130
PLT	-0.083	0.349
IgG	0.282	**0.001**
IgA	0.119	0.179
IgM	0.177	**0.045**
Anti-dsDNA	0.091	0.308
C3	0.063	0.481
C4	-0.041	0.646
ESR	0.317	**<0.001**
CRP	0.132	0.137
RF	0.137	0.125
*β*2-GI	0.065	0.487
24 h proteinuria	-0.074	0.443
SLEDAI-2K	**0.209**	**0.017**

WBC: white blood cell; HB: hemoglobin; PLT: platelet count; IgG: immunoglobulin G; IgA: immunoglobulin A; IgM: immunoglobulin M; anti-dsDNA: anti-double stranded DNA antibodies; C3: complement component 3; C4: complement component 4; ESR: erythrocyte sedimentation rate; RF: rheumatoid factor; *β*2-GPI: anti-*β*2 glycoprotein I Abs; SLEDAI-2K: systemic lupus erythematosus disease activity index 2000.

**Table 3:** Clinical and laboratory characteristics of SLE patients with the elevated and normal levels of serum anti-GAPDH antibodies.

Clinical and laboratory parameters	Anti-GAPDH		*t*/*u*/*χ* ^2^	*P*
	<55.09 (*n* = 44)	≥55.09 (*n* = 86)		
Rash	21 (47.73%)	34 (39.53%)	0.800	0.371
Nervous system	11 (25.00%)	24 (27.91%)	0.125	0.835
Hematological system	26 (59.09%)	59 (68.60%)	1.164	0.281
Joint involvement	15 (34.09%)	41 (47.67%)	2.190	0.139
Malar rash	16 (36.36%)	20 (23.26%)	2.498	0.114
Lung involvement	6 (13.64%)	17 (19.77%)	0.751	0.386
LN	26 (59.09%)	30 (34.88%)	**6.956**	**0.008**
WBC	6.050 (4.20, 7.99)	4.845 (3.56, 7.68)	-1.314	0.189
HB	108.79 ± 20.34	109.29 ± 20.64	0.132	0.895
PLT	178.76 ± 88.07	169.22 ± 85.59	-0.595	0.553
C3	0.62 ± 0.30	0.66 ± 0.30	-0.725	0.470
C4	0.13 (0.07, 0.17)	0.12 (0.06, 0.20)	-0.189	0.850
IgA	2.40 (1.58, 3.73)	2.57 (1.89, 3.87)	-0.688	0.491
IgG	12.85 (9.33, 17.73)	17.10 (13.10, 20.05)	-**0.278**	**0.006**
IgM	0.84 (0.51, 1.23)	1.02 (0.72, 1.49)	-**2.390**	**0.017**
RF	7 (16.67%)	23 (27.06%)	1.683	0.267
CRP	2.75 (1.41, 9.28)	6.65 (2.23, 13.80)	-**2.197**	**0.028**
ESR	21.00 (13.00, 40.00)	35.00 (21.00, 65.50)	-**3.172**	**0.002**
Acl	4.45 (1.73, 11.90)	5.00 (2.10, 10.95)	-0.412	0.680
*β*2-GI	3.84 (2.00, 15.87)	5.47 (2.00, 16.63)	-0.618	0.537
Anti-dsDNA	28.20 (5.80, 153.20)	51.80 (13.50, 200.00)	-1.670	0.095
AnuA	36.07 (5.69, 147.84)	31.45 (7.37, 191.90)	-0.840	0.401
ANA ≥ 1 : 320	35 (79.55%)	75 (87.21%)	0.772	0.380
Anti-SSA	23 (54.76%)	46 (53.49%)	0.018	0.892
Anti-SSB	5 (11.90%)	10 (11.63%)	0.002	1.000
Urine protein	21 (47.73%)	28 (33.33%)	2.532	0.112
SLEDAI-2K ≥ 10	24 (54.55%)	60 (69.77%)	2.950	0.086

LN: lupus nephritis; WBC: white blood cell; HB: hemoglobin; PLT: platelet count; C3: complement component 3; C4: complement component 4; IgA: immunoglobulin A; IgG: immunoglobulin G; IgM: immunoglobulin M; RF: rheumatoid factor; CRP: C-reaction protein; ESR: erythrocyte sedimentation rate; Acl: anti-cardiolipin; *β*2-GPI: anti-*β*2 glycoprotein I Abs; anti-dsDNA: anti-double-stranded DNA antibodies; AnuA: anti-nucleosome antibodies; ANA: anti-nuclear antibodies; anti-SSA: anti-SSA antibody; anti-SSB: anti-SSB antibody; SLEDAI-2K: systemic lupus erythematosus disease activity index 2000.

## Data Availability

The original data supporting our manuscript are data collected from laboratory tests and clinical parameters collected from the recruited patients which did not include any privacy information of the patients. All the data are organized in Excel files and will be available upon application to the Department of Neurology, Peking University Shenzhen Hospital, Shenzhen, China. Our data is available by contacting Jingjing Sun at email sjj1053565474@bjmu.edu.cn or the corresponding author Xiaolin Sun at email sunxiaolin_sxl@126.com.

## Supplementary Materials

Supplementary Materials Figure S1: (a) levels of anti-GAPDH in NPSLE patients with different SLICC-ACR damage index scores. (b) Comparison of SLICC-ACR scores in the NPSLE patients with elevated and normal levels of the serum anti-GAPDH antibodies. SLICC-ACR: Systemic Lupus International Collaborating Clinics/American College of Rheumatology. Figure S2: correlation of the serum antiphospholipid antibodies of NPSLE patients with cerebrovascular lesions and seizure disorders. (a) Correlation of the serum anti-cardiolipin antibodies (Acl) of NPSLE patients with cerebrovascular lesions. (b) Correlation of the serum anti-cardiolipin antibodies (Acl) of NPSLE patients with seizure disorders. (c) Correlation of the serum anti-*β*2 glycoprotein I antibodies of NPSLE patients with cerebrovascular lesions. (d) Correlation of the serum anti-*β*2 glycoprotein I antibodies of NPSLE patients with seizure disorders. (e) Correlation of serum lupus anticoagulant of NPSLE patients with cerebrovascular lesions. (f) Correlation of serum lupus anticoagulant of NPSLE patients with seizure disorders. CVL: cerebrovascular lesions; *β*2-GPI: anti-*β*2 glycoprotein I antibodies. Table S1: correlation of the serum anti-GAPDH antibodies with clinical and laboratory features of NPSLE patients. Table S2: clinical and laboratory characteristics of NPSLE patients with the elevated and normal levels of the serum anti-GAPDH antibodies. Table S3: characteristics of NPSLE and cerebrovascular lesions and seizure disorders in antiphospholipid antibody-positive and negative patients. Table S4: characteristics of NPSLE and cerebrovascular lesions and seizure disorders in Acl antibody-positive and negative patients.
