# Supplementary material for: Anti-GAPDH Autoantibody Is Associated with Increased Disease Activity and Intracranial Pressure in Systemic Lupus Erythematosus
Source: J Immunol Res. 2019 Mar 31;2019:7430780. doi: 10.1155/2019/7430780 (PMC6462327; doi:10.1155/2019/7430780)
Supplement: Supplementary Materials — Figure S1: (a) levels of anti-GAPDH in NPSLE patients with different SLICC-ACR damage index scores. (b) Comparison of SLICC-ACR scores in the NPSLE patients with elevated and normal levels of the serum anti-GAPDH antibodies. SLICC-ACR: Systemic Lupus International Collaborating Clinics/American College of Rheumatology. Figure S2: correlation of the serum antiphospholipid antibodies of NPSLE patients with cerebrovascular lesions and seizure disorders. (a) Correlation of the serum anti-cardiolipin antibodies (Acl) of NPSLE patients with cerebrovascular lesions. (b) Correlation of the serum anti-cardiolipin antibodies (Acl) of NPSLE patients with seizure disorders. (c) Correlation of the serum anti-β2 glycoprotein I antibodies of NPSLE patients with cerebrovascular lesions. (d) Correlation of the serum anti-β2 glycoprotein I antibodies of NPSLE patients with seizure disorders. (e) Correlation of serum lupus anticoagulant of NPSLE patients with cerebrovascular lesions. (f) Correlation of serum lupus anticoagulant of NPSLE patients with seizure disorders. CVL: cerebrovascular lesions; β2-GPI: anti-β2 glycoprotein I antibodies. Table S1: correlation of the serum anti-GAPDH antibodies with clinical and laboratory features of NPSLE patients. Table S2: clinical and laboratory characteristics of NPSLE patients with the elevated and normal levels of the serum anti-GAPDH antibodies. Table S3: characteristics of NPSLE and cerebrovascular lesions and seizure disorders in antiphospholipid antibody-positive and negative patients. Table S4: characteristics of NPSLE and cerebrovascular lesions and seizure disorders in Acl antibody-positive and negative patients. [file 7430780.f1.pdf]

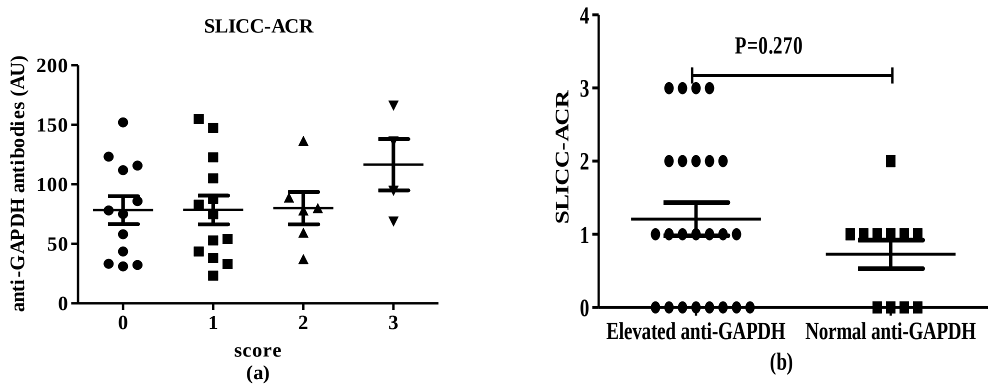


**Supplementary Figure 1**: (a) Levels of anti-GAPDH in NPSLE patients with different SLICC-ACR damage index scores. (b) Comparison of SLICC-ACR scores in the NPSLE patients with elevated and normal levels of serum anti-GAPDH antibodies. SLICC-ACR: Systemic Lupus International Collaborating Clinics/American College of Rheumatology.

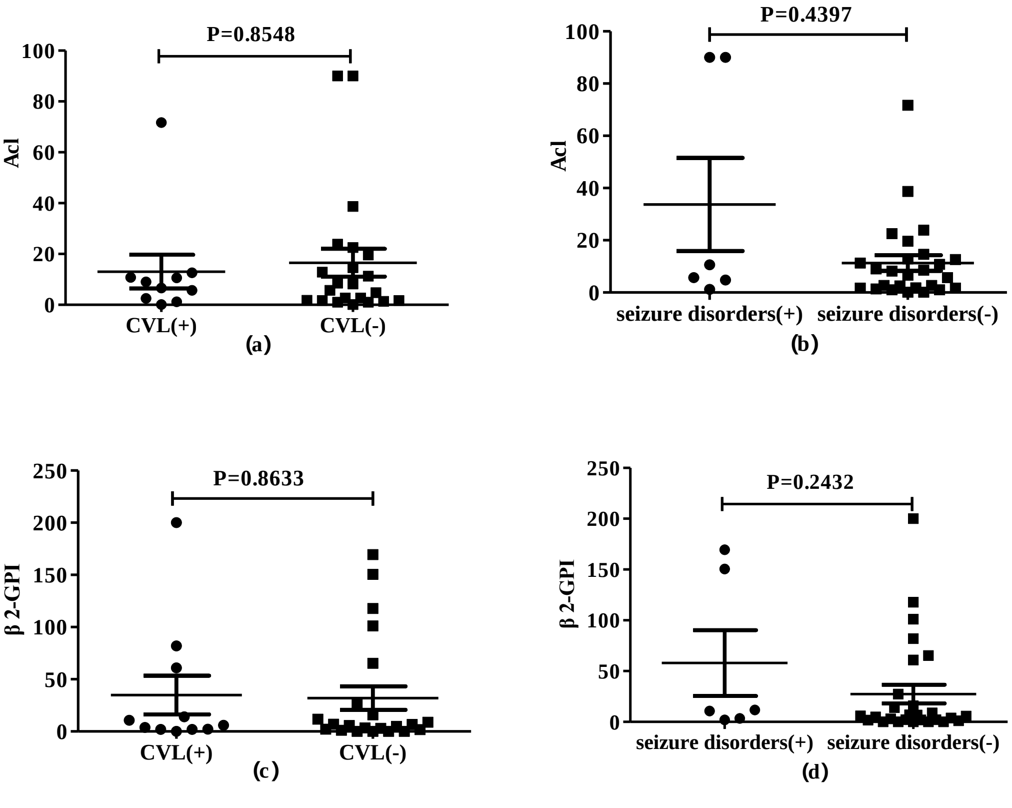

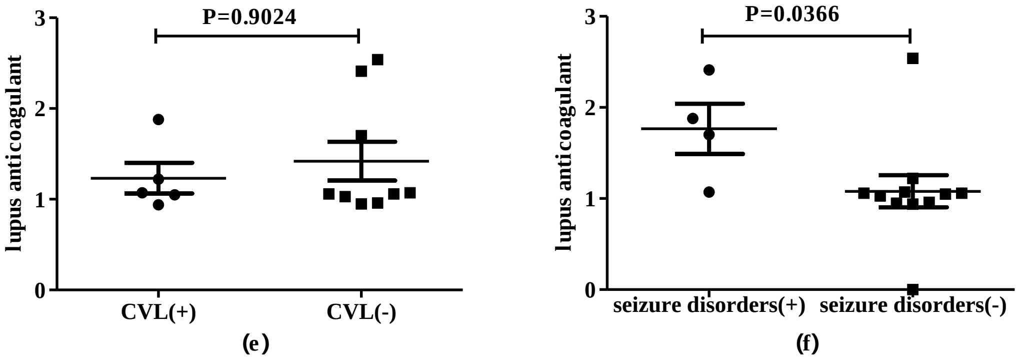


**Supplementary Figure 2:** Correlation of serum antiphospholipid antibodies of NPSLE patients with cerebrovascular lesions and seizure disorders. (a) correlation of serum anti-cardiolipin antibodies (Acl) of NPSLE patients with cerebrovascular lesions. (b) correlation of serum anti-cardiolipin antibodies (Acl) of NPSLE patients with seizure disorders. (c) correlation of serum anti-β2 glycoprotein I antibodies of NPSLE patients with cerebrovascular lesions. (d) correlation of serum anti-β2 glycoprotein I antibodies of NPSLE patients with seizure disorders. (e) correlation of serum lupus anticoagulant of NPSLE patients with cerebrovascular lesions. (f) correlation of serum lupus anticoagulant of NPSLE patients with seizure disorders.

CVL: cerebrovascular lesions; β2-GPI : anti-β2 glycoprotein I antibodies.

## Supplementary Table 1: Correlation of serum anti-GAPDH antibodies with clinical and laboratory features of in NPSLE patients.

| Clinical manifestations and laboratory features | anti-GAPDH | |
|---|---|---|
| | Spearman r | P |
| Age | 0.112 | 0.524 |
| WBC | -0.039 | 0.825 |
| HB | -0.163 | 0.349 |
| PLT | -0.144 | 0.410 |
| IgG | 0.077 | 0.666 |
| IgA | 0.119 | 0.502 |
| IgM | 0.306 | 0.078 |
| Anti-dsDNA | 0.106 | 0.549 |
| anti-Sm | -0.143 | 0.420 |
| Anti-SSA | 0.040 | 0.821 |
| Anti-SSB | -0.322 | 0.063 |
| C3 | 0.228 | 0.194 |
| C4 | 0.025 | 0.887 |
| ESR | 0.323 | 0.066 |
| CRP | 0.222 | 0.207 |
| RF | -0.164 | 0.355 |
| Acl | -0.022 | 0.903 |
| Lupus anticoagulant | -0.043 | 0.879 |
| β2-GI | 0.157 | 0.382 |
| 24h proteinuria | -0.171 | 0.348 |
| SLEDAI-2K | 0.247 | 0.153 |

| | | |
|---|---|---|
| Intracranial pressure | **0.567** | **0.004** |
| Chloride in CSF | 0.040 | 0.829 |
| Glucose in CSF | -0.047 | 0.799 |
| Protein in CSF | -0.152 | 0.410 |

WBC: white blood cell; HB: hemoglobin; PLT: platelet count; IgG: Immunoglobulin G; IgA: Immunoglobulin A; IgM: Immunoglobulin M; Anti-dsDNA: Anti-double stranded DNA antibodies; Acl: anti-cardiolipin; anti-Sm: anti-Smith; Anti-SSA: anti-SSA antibody; Anti-SSB: anti-SSB antibody; C3: component3; C4: component 4; ESR: Erythrocyte sedimentation rate; CRP: C-reaction protein; RF: rheumatoid factor; β2-GPI: anti-β2 glycoprotein I Abs; SLEDAI-2K: Systemic Lupus Erythematosus Disease Activity Index 2000; CSF: cerebrospinal fluid.

**Supplementary Table 2: Clinical and laboratory characteristics of NPSLE patients with the elevated and normal levels of serum anti-GAPDH antibodies.**

| Clinical and laboratory parameters | anti-GAPDH | | $t/u/\chi^2$ | P |
|---|---|---|---|---|
| | ＜55.09 (n=11) | ≥55.09 (n=24) | | |
| **NPSLE manifestations** | | | | |
| Headache | 5/11(45.45%) | 16/24(66.67%) | 1.414 | 0.234 |
| CVL | 1/11(9.09%) | 11/24(45.83%) | **4.520** | **0.034** |
| Seizure disorders | 4/11(36.36%) | 2/24(8.3%) | **4.172** | **0.041** |
| Cognitive dysfunction | 2/11(18.18%) | 4/24(16.67%) | 0.012 | 0.912 |
| Acute confusional state | 0 | 2/24(8.3%) | - | - |
| Demyelinating syndrome | 1/11(9.09%) | 1/24(4.17%) | - | 0.536 |
| Autonomic disorder | 1/11(9.09%) | 2/24(8.3%) | - | 1.000 |
| Psychosis | 0 | 2/24(8.3%) | - | - |
| Mood disorder | 0 | 1/24(4.17%) | - | - |
| Mononeuropathy, | 1/11(9.09%) | 0 | - | - |
| single/multiplex | 0 | 2/24(8.3%) | - | - |
| Neuropathy, cranial | | | | |
| LN | 6/11(54.55%) | 13/24(54.17%) | 0 | 0.983 |
| D-Dimer | 5/10(50.0%) | 15/24(62.5%) | 0.455 | 0.500 |
| IgA | 2.995±1.609 | 2.791±1.481 | -0.364 | 0.718 |
| IgG | 17.72±7.883 | 17.87±4.693 | 0.065 | 0.948 |
| IgM | 0.584(0.483,1.340) | 0.92(0.665,1.36) | -1.436 | 0.151 |
| C3 | 0.50±0.15 | 0.62±0.31 | 1.567 | 0.127 |
| C4 | 0.11±0.05 | 0.11(0.06,0.01) | -0.460 | 0.645 |
| CRP | 3.46(1.35,11.5) | 7.80(3.18,19.5) | -0.834 | 0.404 |
| ESR | 31.89±26.48 | 53.83±28.18 | 2.023 | 0.052 |

| | | | | |
|---|---|---|---|---|
| Acl | 4.80(1.70,22.50) | 8.50(2.10,13.75) | -0.437 | 0.662 |
| Anti-dsDNA | 18.5(0.00,168.6) | 93.4(24.6,236.8) | -1.844 | 0.065 |
| ANUA | 69.62(8.88,108.7) | 77.03(10.65,179.7) | -0.958 | 0.338 |
| ANA≥1:320 | 9/10(90.00%) | 16/24(66.67%) | 1.975 | 0.160 |
| 24h-Urine protein | 0.76(0.165,1.338) | 0.385(0.125,1.198) | -0.732 | 0.464 |
| SLEDAI-2K≥10 | 23.00±7.39 | 25.58±7.99 | 0.877 | 0.387 |

CVL: cerebrovascular lesions; LN: lupus nephritis; IgA: Immunoglobulin A; IgG: Immunoglobulin G; IgM: Immunoglobulin M; C3: component3; C4: component 4; CRP: C-reaction protein; ESR: Erythrocyte sedimentation rate; Acl: anti-cardiolipin; Anti-dsDNA: Anti-double stranded DNA antibodies; ANUA: Anti-nucleosome antibodies; ANA: Anti-nuclear antibodies; SLEDAI-2K: Systemic Lupus Erythematosus Disease Activity Index 2000.

**Supplementary Table 3: characteristics of NPSLE and cerebrovascular lesions and seizure disorders in anti-phospholipid antibodies positive and negative patients.**

| | anti-phospholipid antibodies(+) | anti-phospholipid antibodies(-) | $\chi^2$ | P |
|---|---|---|---|---|
| NPSLE | 15/33（45.45%） | 20/82（24.39%） | 1.541 | 0.215 |
| CVL | 5/15（33.33%） | 5/18（27.78%） | - | 1.000 |
| Seizure disorders | 4/15（26.67%） | 2/18（11.11%） | - | 0.375 |

CVL: cerebrovascular lesions

**Supplementary Table 4: characteristics of NPSLE and cerebrovascular lesions and seizure disorders in Acl antibodies positive and negative patients.**

| | Acl (+) | Acl (-) | $\chi^2$ | P |
|---|---|---|---|---|
| NPSLE | 11/29（37.93） | 21/91（23.08） | 2.481 | 0.115 |
| CVL | 3/11（27.27%） | 7/21（33.33%） | - | 1.000 |
| Seizure disorders | 3/11（27.27%） | 3/21（14.29%） | - | 0.390 |

CVL: cerebrovascular lesions; Acl: anti-cardiolipin antibodies.
